# Clinical and genetic aspects of KBG syndrome

**DOI:** 10.1002/ajmg.a.37842

**Published:** 2016-09-26

**Authors:** Karen Low, Tazeen Ashraf, Natalie Canham, Jill Clayton‐Smith, Charu Deshpande, Alan Donaldson, Richard Fisher, Frances Flinter, Nicola Foulds, Alan Fryer, Kate Gibson, Ian Hayes, Alison Hills, Susan Holder, Melita Irving, Shelagh Joss, Emma Kivuva, Kathryn Lachlan, Alex Magee, Vivienne McConnell, Meriel McEntagart, Kay Metcalfe, Tara Montgomery, Ruth Newbury‐Ecob, Fiona Stewart, Peter Turnpenny, Julie Vogt, David Fitzpatrick, Maggie Williams, DDD Study, Sarah Smithson

**Affiliations:** ^1^ University Hospitals Bristol NHS Trust/University of Bristol Bristol United Kingdom; ^2^ Guy's and St Thomas’ NHS Trust London United Kingdom; ^3^ North West Thames Regional Genetics Service Harrow London United Kingdom; ^4^ Manchester Centre For Genomic Medicine St Mary's Hospital Manchester United Kingdom; ^5^ Institute of Human Development University of Manchester Manchester United Kingdom; ^6^ Teesside Genetics Unit The James Cook University Hospital Middlesbrough United Kingdom; ^7^ Wessex Clinical Genetics Service Southampton United Kingdom; ^8^ Liverpool Women's NHS Trust Liverpool United Kingdom; ^9^ Genetic Health Service NZ Christchurch Hospital Christchurch New Zealand; ^10^ Genetic Health Service NZ Auckland Hospital Auckland New Zealand; ^11^ Bristol Genetics Laboratory North Bristol NHS Trust Bristol United Kingdom; ^12^ West of Scotland Department of Clinical Genetics Glasgow United Kingdom; ^13^ Royal Devon and Exeter Hospital Exeter United Kingdom; ^14^ Northern Ireland Regional Genetics Service Belfast City Hospital Belfast Ireland; ^15^ South West Thames Clinical Genetics Service St Georges Hospital London United Kingdom; ^16^ Northern Genetics Service Newcastle Upon Tyne United Kingdom; ^17^ West Midlands Regional Genetics Service Birmingham United Kingdom; ^18^ MRC Human Genetics Unit MRC Institute of Genetics and Molecular Medicine University of Edinburgh Edinburgh United Kingdom; ^19^ Wellcome Trust Sanger Institute Cambridgeshire United Kingdom

**Keywords:** KBG syndrome, macrodontia, *ANKRD11*

## Abstract

KBG syndrome is characterized by short stature, distinctive facial features, and developmental/cognitive delay and is caused by mutations in *ANKRD11*, one of the ankyrin repeat‐containing cofactors. We describe 32 KBG patients aged 2–47 years from 27 families ascertained via two pathways: targeted *ANKRD11* sequencing (TS) in a group who had a clinical diagnosis of KBG and whole exome sequencing (ES) in a second group in whom the diagnosis was unknown. Speech delay and learning difficulties were almost universal and variable behavioral problems frequent. Macrodontia of permanent upper central incisors was seen in 85%. Other clinical features included short stature, conductive hearing loss, recurrent middle ear infection, palatal abnormalities, and feeding difficulties. We recognized a new feature of a wide anterior fontanelle with delayed closure in 22%. The subtle facial features of KBG syndrome were recognizable in half the patients. We identified 20 *ANKRD11* mutations (18 novel: all truncating) confirmed by Sanger sequencing in 32 patients. Comparison of the two ascertainment groups demonstrated that facial/other typical features were more subtle in the ES group. There were no conclusive phenotype–genotype correlations. Our findings suggest that mutation of *ANKRD11* is a common Mendelian cause of developmental delay. Affected patients may not show the characteristic KBG phenotype and the diagnosis is therefore easily missed. We propose updated diagnostic criteria/clinical recommendations for KBG syndrome and suggest that inclusion of *ANKRD11* will increase the utility of gene panels designed to investigate developmental delay. © 2016 The Authors. *American Journal of Medical Genetics Part A* Published by Wiley Periodicals, Inc.

## INTRODUCTION

KBG syndrome (MIM # 148050) [Herrmann et al., 1975] combines short‐stature, macrodontia of the permanent central upper incisors, distinctive facial features, learning difficulties, and neurobehavioral problems [Herrmann et al., 1975; Smithson et al., 2000; Skjei et al., 2007; Sirmaci et al., 2011; Youngs et al., 2011]. Other recognized features include seizures, cardiac abnormalities, and hearing loss [Brancati et al., 2006; Youngs et al., 2011; Ockeloen et al., 2015]. Heterozygous mutations in *ANKRD11* (ankyrin repeat domain‐containing protein 11) were shown to cause KBG syndrome [Sirmaci et al., 2011]. Deletions of 16q24 including *ANKRD11* were reported to lead to a similar phenotype [Willemsen et al., 2010; Isrie et al., 2012; Sacharow et al., 2012]. In 2006, the total number of cases reported worldwide was 45 [Brancati et al., 2006], but the advent of whole exome sequencing (ES) has increased recognition, including recent reports of over 25 new KBG patients [Ockeloen et al., 2015; Walz et al., 2015].

We established single gene analysis of *ANKRD11* (TS) as a diagnostic test at Bristol Genetics Laboratory in 2014 for patients considered to have KBG syndrome. Concurrently, the Deciphering Developmental Disorders study utilized ES to identify diagnoses for children with developmental delay and additional features [Wright et al., 2015]. Here, we describe clinical and molecular genetic findings in 31 previously unreported KBG patients and one adult who was previously reported as a child [Smithson et al., 2000], ascertained through both sources. We compare the phenotypic characteristics in each of our ascertainment groups and with patients reported by other authors. We alert clinicians to the key features of KBG syndrome including a wide fontanelle, which may be the first indication that an infant is affected. Data on older patients is presented, providing insight into the long‐term outcome of the condition. Recognition of this diagnosis in young adults has important implications for genetic counselling.

## METHODS

Patients were ascertained either via TS or ES [Wright et al., 2015] as above. In the TS group, a Clinical Geneticist in the UK (or New Zealand in one case) had suspected the diagnosis of KBG syndrome based on clinical features and sent a DNA sample to Bristol Genetics Laboratory for *ANKRD11* analysis. Consent for genetic testing was obtained at the time of sample collection. The ES group of children had been recruited to the DDD study because no clinical diagnosis had been made (20 patients from 10 of the 23 UK Clinical Genetic Centers). These children had a history of developmental delay/intellectual disability with or without additional features. DNA samples were collected as parent/child trios. Array CGH and ES were performed looking for copy number variation (CNV) and de novo variants. Variants were interrogated on the basis of link to phenotype and using standard in silico tools [Wright et al., 2015]. A DDD‐approved complementary analysis project (CAP) allowed us to access the exome data and phenotypes of all subjects identified with *ANKRD11* variants through the DDD Decipher website [Firth et al., 2009]. For both groups, clinicians responsible for the patients’ care were approached. Patient consent and phenotyping was initially undertaken by the referring clinician and reviewed by KL and SS. A deep phenotyping questionnaire was devised based on current literature. Broad subheadings in the questionnaire included growth, development, learning, vision and hearing, neurobehavioral, skeletal, dental, cardiac, gastrointestinal, immune, and other features. We paid particular attention to learning difficulties and behavioral phenotype which are often significant in KBG syndrome [Lo‐Castro et al., 2013].

Genomic DNA was received from an external laboratory or isolated from peripheral blood leukocytes using a Gentra Puregene cell kit (QIAGEN Ltd). Coding regions of the *ANKRD11* gene (13 exons including intron/exon boundaries extending to the branch sites) were amplified in 27 fragments using a MegaMix (MicroZone). Primers were designed to GenBank Reference Sequence NM_013275.5 using Primer3 software [Koressaar and Remm, 2007; Untergasser et al., 2012]. The large size of exon 9 (>6 kb of coding sequence) required the design of 17 overlapping sequencing amplicons in order to cover the whole exon. The high GC content of the *ANKRD11* gene necessitated the use of a GC‐RICH (Roche) PCR system. Primer sequences are available on request.

M13 tagged bidirectional sequencing was undertaken using a BigDye Terminator v3.1 Cycle Sequencing Kit and 3730 DNA Genetic Analyzer (Applied Biosystems) with Mutation Surveyor DNA Variant Analysis Software v3.97 (Softgenetics). Alamut software v2.3.1 (Interactive Biosoftware, Rouen, France) was used to predict the effect of genetic variation. The software integrates PolyPhen‐2, Align GVGD and SIFT and five splice site prediction programs: SpliceSiteFinder, MaxEntScan, Human Splice Finder, NNSPLICE, and GeneSplicer. All DDD results were confirmed by Sanger sequencing either in our laboratory or locally.

## RESULTS

We studied 32 patients from 27 families in total. Thirteen patients were ascertained through our Bristol laboratory and for 12 (11 from the UK and one New Zealand) TS was undertaken. One additional patient had a microdeletion of 16p24.3 including *ANKRD11* identified by array CGH but was excluded from the study. Four patients were from one family (a father and three daughters). Twenty patients were ascertained through DDD including one family (a mother, son, and daughter).

**Figure 1:**
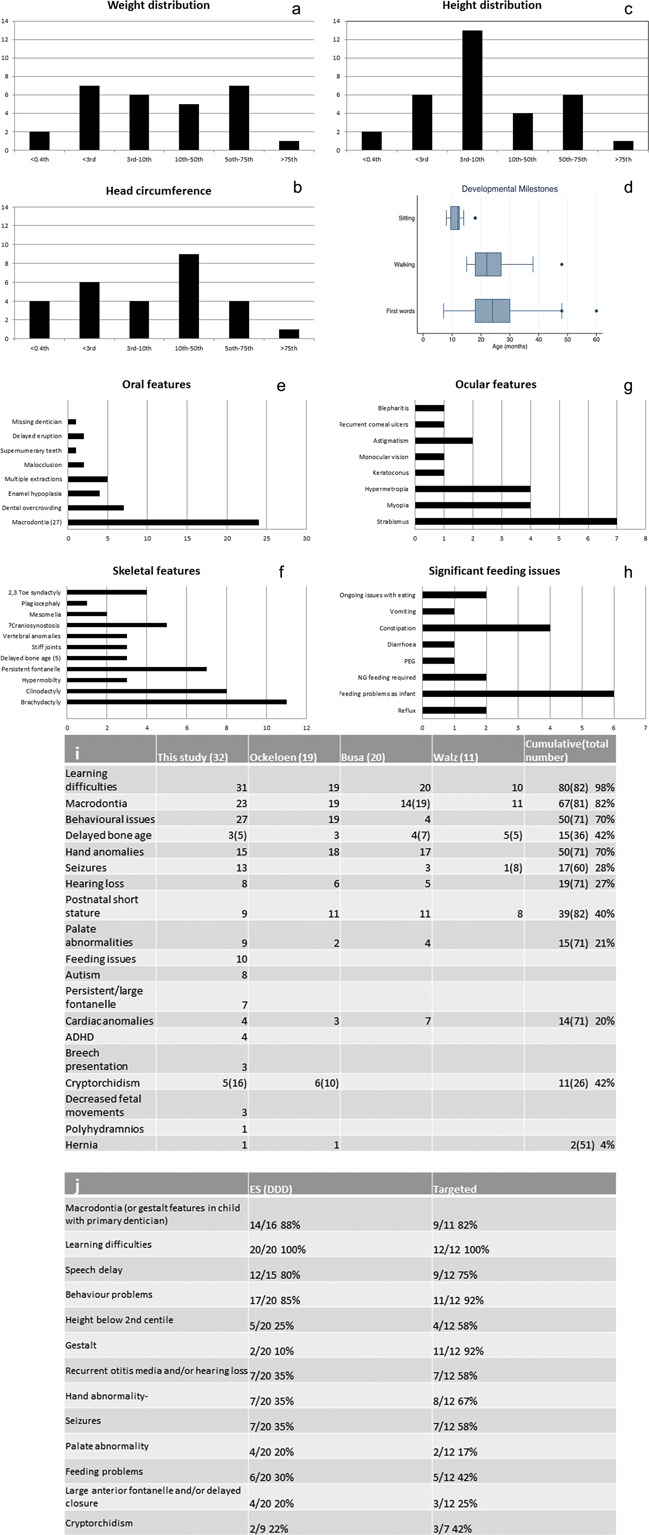
Clinical features of KBG syndrome. (**a–c**) Bar charts show number of patients in each centile category for weight, head circumference, and height. (**d**) Box and whisker plot indicates age in months at which patients were first able to sit, walk, and speak first words (dots denote statistical outliers). (**e–h**) Bar charts demonstrate numbers of patients showing specific oral, skeletal, ocular anomalies, and feeding difficulties. (**i–j**) Tables compare the frequency of specified features in patients our cohort compared with cumulative data (ours and previously reported cases) and the frequency of features in the two ascertainment groups. In e,f, and i the bracketed numbers denotes the total number of patients in which the feature/data could be sought. (**i**)Cumulative data from Ockeloen et al. [2015]; Busa et al. [2015]; and Walz et al. [2015].

All but two patients consented to clinical photographs. In our analysis of the clinical findings of KBG we initially considered our cohort separately. We then amalgamated our data with those previously published which enabled us to estimate the frequency of features in 100 patients (Fig. 1i and j). The wide age range in our cohort provided some insight into the developmental outcome and prognosis in KBG syndrome.

### Facial Features

**Figure 2:**
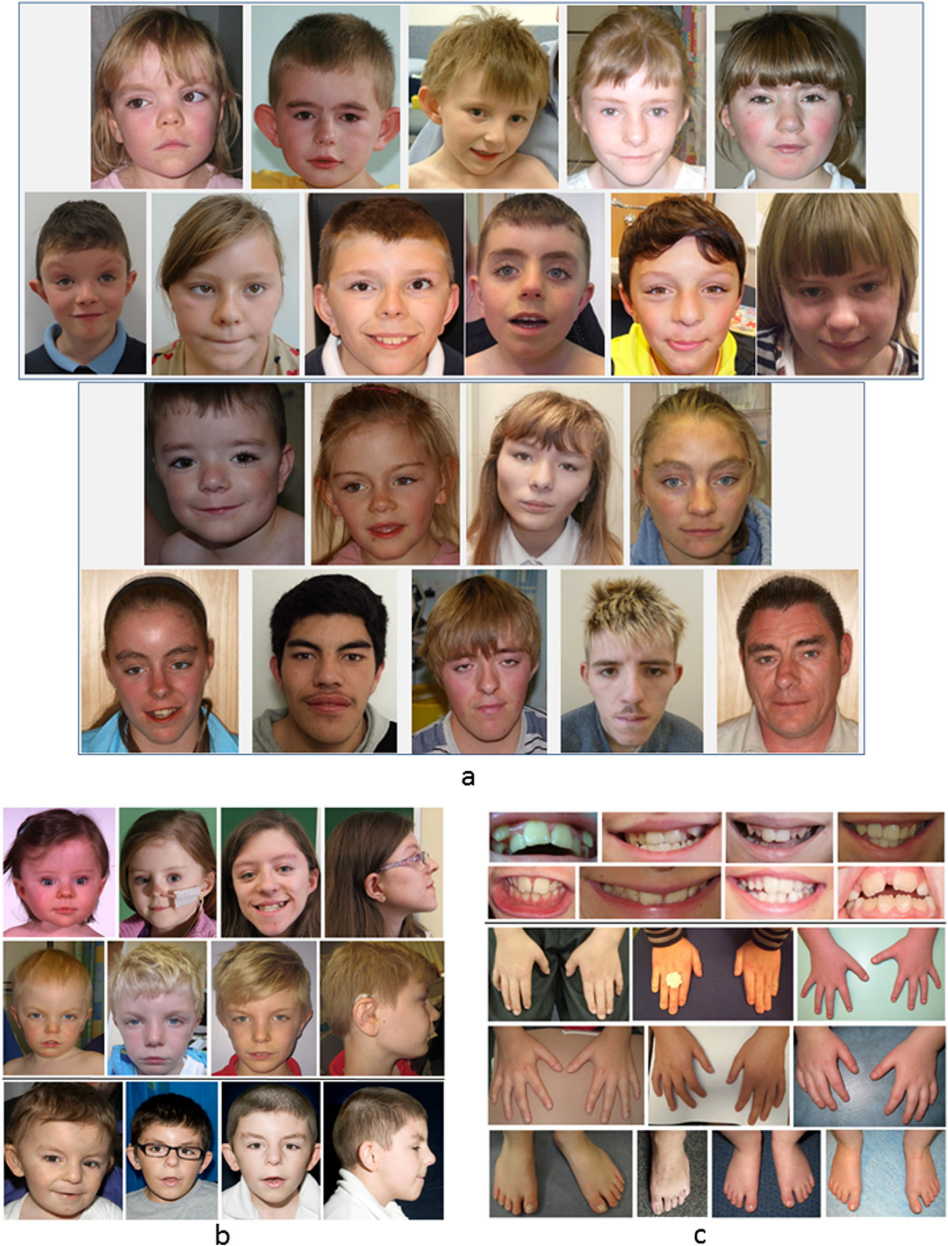
Facial, dental and limb features in KBG syndrome. (**a**) Facial features of patients with KBG syndrome during infancy, childhood, and targeted testing (TS, below). (**b**) Evolution of the facial phenotype and profile of 3 patients with KBG syndrome during childhood and adolescence ascertained through TS (2 patients above) and TS (1 patient below). (**c**) Secondary dentition of patients with KBG showing macrodontia of central incisors, dental crowding, and extra teeth in some cases. The hands and feet show subtle brachydactyly and clinodactyly of fifth fingers. [Color figure can be viewed at http://wileyonlinelibrary.com].

The facial features of our patients with KBG syndrome are shown in Figure 2a and b (ascertained through ES above and TS below). Subjective appraisal of the images confirmed the presence of characteristic facial features in some but not all patients. These included broad triangular face, short neck, wide or bushy eyebrows, often with synophrys, hypertelorism, prominent ears or dysplastic helices, bulbous nasal tip, prominent nasal bridge, long and smooth philtrum, and thin upper lip. We found that patients in the TS group were more likely to express several of these features. A few patients in the ES group could not be identified as having KBG syndrome on the basis of their facial features.

### Growth

The parameters of growth in our patients (Fig. 1a–c) are in keeping with the other reported cohorts [Kim et al., 2015; Ockeloen et al., 2015] and cumulative data showed the frequency of short stature (height <3rd centile) was 40% (Fig. 1i).

### Perinatal Period

Few complications occurred in pregnancy and most 20‐week anomaly scans were normal apart from single instances of a single umbilical artery, an atrio‐ventricular septal defect (AVSD), placenta praevia, and decreased fetal movements. All babies reached a gestation of at least 36 weeks and the vast majority were delivered at term. Only two babies were breech presentation. Birthweights were all within normal parameters (2nd–98th centiles). Nineteen percent (6/32) of babies were reported to have significant feeding difficulties after birth with some requiring nasogastric tube‐feeding (Fig. 1h).

### Developmental Progress/Learning Difficulties

The ages at which milestones were reached was delayed (Fig. 1d), especially speech; most patients used their first single words between 2 and 3 years of age and one patient had only a few words aged 4. All children achieved walking and the oldest to do so was aged 3 years and 2 months. In no instances did patients lose skills or regress. Four patients attended a school for children with special needs and four went to a mainstream school but within a specified special needs unit (two within an autism unit). The rest attended mainstream school usually with a statement of educational needs and received variable one to one support within the classroom. Across the literature, 99% of patients had learning difficulties (Fig. 1i), but they could be very mild.

### Neurodevelopment/Behavior

Fourteen patients (43%) had seizures although the age of onset varied from infancy to mid‐teens. Seizure type was heterogeneous and included absences, grand mal, myoclonic jerks, and nocturnal seizures. Some patients without a diagnosis of epilepsy had staring episodes, infantile seizures, or febrile fits which resolved. Brain MRI scans were available in 13 children; 2 showed single abnormalities including mild periventricular leukomalacia with normal ventricles and an isolated dilated left ventricle. Two scans showed moderate enlargement of the cisterna magna with normal ventricles (one also showing atrophic changes). Only four children had a movement disorder including one with Tourette‐like tics. One adult had a wide‐based gait with apparent foot drop and involuntary flailing of the arms on walking. Eight children (25%) had a formal diagnosis of autistic spectrum disorder (ASD) and one more had some autistic features. Five patients had a diagnosis of attention deficit hyperactivity disorder (ADHD) and three others had mild symptoms in this spectrum.

Behavioral issues occurred in all but two of the patients; however, their nature was vary variable and in some cases mild. There were recurring themes, such as poor concentration, lack of attention span, and restless movement. Some patients had obsessions and could become fixated on a favorite object or routine or disliked change with behavior deteriorating at times of stress. Anxiety and shyness were reported in several patients associated with difficulty in understanding social situations and making friendships. In contrast, some parents noted a lack of stranger‐awareness and disinhibition in their children when interacting with others. Some patients were described as having a low anger threshold and a few had severely challenging behavior such as tantrums, inconsolable upset, and occasional aggressive outbursts. One patient had a diagnosis of oppositional defiant disorder. Other problems included a sensory processing disorder, rocking and flicking of elastic bands, a very unusual phenotype of echolalia‐imitation of an American accent, and autistic‐type behavior. Sleep disturbances were reported in six patients and the majority were resistant to melatonin. Two further patients had severe sleep disruption as babies which resolved with age. One child had sleep apnea which persisted after adenotonsillectomy.

### Ear, Nose, Throat, and Oral Cavity

The key dental findings in our study (Figs. 1e and 2c) included macrodontia^1^ of upper incisors in most of our patients (23/27 with secondary dentition) and 82% of patients with KBG syndrome overall (Fig. 1i). Palatal abnormalities occurred in a quarter of patients (8/32); four had sub‐mucous cleft palate, two had bifid uvulas, one had velopharyngeal incompetence and one had a short palate necessitating two pharyngoplasties. Two further patients had hyper‐nasal speech, but neither was formally assessed by a palate team nor had a comprehensive examination under anaesthetic, which was the case for the majority of patients. We may therefore have underestimated the frequency of palatal abnormalities.

Permanent conductive hearing loss of varying degrees was reported in a quarter of patients (8/32), all of whom had a history of recurrent otitis media and/or tympanic membrane perforation treated with grommet insertion. A further seven reported recurrent middle ear infections without lasting hearing loss. One patient required tympanic membrane grafts at the age of 13 and another had sensorineural hearing loss in the context of a positive maternal family history.

### Skeleton

The most consistent findings were in the distal limbs including brachydactyly especially of the 5th fingers with striking clinodactyly (Fig. 1f). Hand function was normal. The feet were also affected with brachydactyly; often with wide spaces between the 1st and 2nd toes. Interestingly seven (22%) of patients had a large anterior fontanelle with delayed closure; in one patient it still remains patent aged 4.5 years. Ockeloen et al. [2015] reported the same finding in patient 6 of their cohort. A further two patients were reported to have a very large fontanelle as a baby, but this did not persist. A skeletal survey was available in six patients and three of these revealed significant but non‐specific radiological signs. The first showed a copper‐beaten appearance of the skull with small anterior beaks on the vertebrae at the thoracolumbar junction. Another showed radial‐head dislocation with a wide carrying angle at the elbow, short distal phalanges, and a thoracic kyphosis. The third showed shortening of the pedicles of the lumbar vertebrae, brachycephaly, and a relatively long fibula on the side examined. A fused metopic suture was reported in one patient.

### Gastrointestinal System

Gastrointestinal problems were seen in several patients. Feeding difficulties were the most notable affecting eight patients. These started in the neonatal period and resolved in some cases (Fig. 1h) but continued in three cases. One girl, who failed to thrive required nasogastric feeding until the age of 4 years and her weight remained below the 0.4th centile at 13 years. One boy was unable to feed orally at the age of 4.5 years and is dependent on a gastrostomy tube (he also has a sensory processing disorder and autism). Gastro‐oesophageal reflux and severe constipation were also observed. One adult has a life‐long history of diarrhea and an abnormal lack of thirst which has resulted in two hospital admissions for dehydration. One requires a patch for continual drooling.

### Other Aspects of Phenotype

Half the patients (17/32) had eye abnormalities, most commonly strabismus and refractive errors (Fig. 1g). Congenital heart abnormalities occurred in 4/32 (13%), including a patent ductus arteriosus, an AVSD and two patients had small VSDs. One further patient had a structurally normal heart but second degree heart block on ECG (aged 6).

Many patients displayed mild hypertrichosis with thick hair, full eyebrows with lateral interruptions and mild synophrys (Fig. 2a and b). Skin abnormalities were found in several patients. One had a speckled hyperpigmented patch over his inguinal region. He also had two hair whorls on left and right sides of the occiput and a low V‐shaped posterior hairline. One patient had a large hyperpigmented patch on his left arm and another had an asymmetric hair line, a tendency to skin bruising, and delayed wound healing. One had keloid scarring with rapid‐growing thick hair and nails. Another had generalised ichthyosis and predominantly lower limb lymphoedema. Cutis marmorata was described in one patient. Dystrophic nails, particularly on the toes, occurred in 6/32 patients. Recurrent infections (largely respiratory) were described in 6/32 patients although immune function studies did not detect any specific deficit. Five patients had surgery for cryptorchidism and one also had an inguinal hernia repair.

### Adulthood

Eight of the cohort were aged 18 or over including a parent identified after his daughter was diagnosed. He reported slow educational progress at primary school and did not achieve qualifications but was living independently as a father of three with no external help. Similarly, a mother diagnosed after KBG syndrome was identified in her son through DDD, described mild learning difficulties but was living independently, working in a kitchen and looking after her family without support. Her son achieved a few GCSE‐level qualifications and was living in his own accommodation with some help from social services. The other five young adults were all living with their parents who considered it was unlikely they would be able to live alone, although they had many skills such as being able to operate computers. Two were embarking on college courses at the time of the study.

### Genotype–Phenotype Correlations

Within our cohort, we identified 20 different *ANKRD11* mutations in 32 patients with KBG syndrome (Supplementary Table SI). All mutations reside within exon 9, consistent with it containing >80% of the coding sequence and are predicted to cause truncation of the ANKRD11 protein concurring with the mechanism of pathogenicity previously described [Walz et al., 2015]. Of the 20 mutations we identified, 18 were previously unreported. Previously described mutations were found in eight patients (four from the same family) with c.1903_1907del [Ockeloen et al., 2015; Parenti et al., 2016] (Table Ia) and one patient with c.2398_2401del [Walz et al., 2015]. Novel recurrent mutations identified in this study included a deletion in three patients, c.2408‐2412del and a nonsense mutation, c.1801C>T, p.(Arg601*) in two patients (Table Ib). The additional novel mutations occurred in single patients. Comparison of the phenotypes of all reported cases of recurrent mutations did not highlight specific correlations, although we noted that none of the eight c.1903_1907del patients had hearing loss or severe otitis media which was common in the cohort overall.

**Table I:** Recurrent *ANKRD11* Mutations

Recurrent *ANKRD11* mutations c.1903_1907del, p.(Lys635Glnfs^*^26)^a^											
Patient	19 (9y 6m) M DDD	26 (9y 9m) F	4 (13y 3m) M DDD	5 (21y) F^*^	6 (19y) F^*^	7 (12y) F^*^	8 (47y) M^*^	33 (3y 3m) M DDD	Ockeloen et al. [2015] pt 6 (38y) F	Ockeloen et al. [2015] pt 10 (11y) M	Walz et al. [2015] pt B (13y) M
Macrodontia/other dental abnormalities	+; overcrowding	−	+; EH; overcrowding	+	+; overcrowding	+	+	− (primary dentician)	+	+; additional dental problems	+
Short stature	+	−	−	+	+	−	−	−	+	− (on GH)	+
Learning difficulties	+; mainstream school	+; SEN 15hrs/wk	SEN 25 hr 1:1	Moderate	No qualifications, limited literacy	Moderate	Some concerns raised at school	SEN; mainstream school	Moderate	Mild; dyslexia	+
Skeletal abnormalities	−	−	−	2, 3 toe syndactyly	−	−	Noted to have small feet UK size 6	Bilateral cervical ribs	+	+	+
Delayed bone age	−	−	−	−	−	−	−	−	−	+	+
Hand anomalies	−	+	−	+	+	+	+	−	+	+	+
Ocular	−	+; blepharitis	−	—	−	−	−	−	−	−	−
Hearing Loss	−	−	−	—	−	−	−	−	−	−	unknown
Neurological	−	+; lamotrigine	Nocturnal seziures	Seizures	−	Seizures	−	−	−	Poor short term memory	−
Behavioural abnormalities	Anxiety, behaviour worse when stressed	“lovely behaviour”	Anxious	Yes, unspecified	Severe–tantrums, oppositional defiant disorder	Yes, toileting issues	−	Problems with attention and concentration	Compulsive behaviour	Temper tantrums, impaired communication skills	unknown
Palate	−	−	+	—	−	−	−	−	−	assymetric	unknown
Heart	−	Early VSD	AVSD	—	−	−	−	−	−	−	unknown
Other		Hypermobile in some joints	Repetitive movements	Dysplastic 3,4,5 toenails, indented earlobes	DCF	Severe constipation	−	Cryptorchidism, continual drooling;	Large fontanelle at birth	Slightly enlarged ventricles	−

Recurrent *ANKRD11* mutations c.2408_2412 del, p.(Lys803Argfs^*^5)^b^						
Patient	1 (6y 4m) F DDD	18 (6y 10m) M DDD	20 (13y 3m) F DDD	13 (17y) F DDD	27 (23y 1m) M	
Macrodontia/other dental abnormalities	Multiple extractions	+	+; delayed eruption	−; malocclusion	+	
Short stature	+ (<0.4th)	+	−	−	+	
Learning difficulties	Maximum SEN	+	+; SEN, moderate ID	some extra help at school	+; SEN; no qualifications	
Skeletal abnormalities	+	+	+	+	−	
Delayed bone age	+	+	−	+	+ aged 7	
Hand anomalies	+	+	+	+	+	
Ocular	+	Corneal ulcers	+;	−	+	
Hearing Loss	+; TM perforations; x1grommet; hearing aid	−	−	−	Bilat grommets	
Neurological	Mild PVL	−	+; abscences	−	Movement disorder	
Behavioural abnormalities	Autistic like	Sensory processing disorder, “challenging”	Autistic like; obsessions	−	Challenging; no social awareness, outbursts. Stress/anxiety	
Palate	—	−	−	−	−	
Heart	—	−	Small apical VSD	−	−	
Other	DCF;ant. placed anus	Fused metopic suture, cryotrchidism	Severe feeding issues; infections, lacrimal duct surgery; pain insensitivity,	Puffy feet as neonate	Hypermobile elbows. Keloid scarring.	

SEN, statement of education need; PVL, periventricular leukomalacia on MRI; ant, anterior; AF, anterior fontanelle; dn, de novo; DCF, delayed closure of fontanelle age >3y; EH, enamel hypoplasia; AF, anterior fontanelle; TM, tympanic membrane; SNHL(mat), sensorineural hearing loss (maternal history); SPC, single palmar crease.

Skeletal abnormalities: costovertebral abnormalities; postnatal short stature, <2nd centile. Hand abnormalities to include—brachydactyly, 5th finger clinodactyly. Ocular abnormalities = strabismus, hypermetropia, astigmatism; myopia. Palate to include: cleft lip and or palate; submucous cleft, velopharyngeal insufficiency; bifid uvula.

If investigations have not been done designated as “−”.

^*^ Patient 5–8 members of one family. **Patient 29–31 members of one family.

^a^ Comparison of phenotypic findings in seven patients with recurrent c.1903_1907del (5–7 are daughters of eight) with those reported with the same mutation [Ockeloen et al., 2015; Walz et al., 2015].

^b^ Comparison of phenotypic findings in three patients with c.2408_2412del and in two patients with c.1801C>T.

In addition to the patients in this study, we have analyzed additional samples for patients who were referred for TS or for validation after DDD. In this group an additional six mutations have been identified, two of which are of interest because they are outside exon 9. The first, a de novo frameshift in exon 6, occurred in a patient (DDD266108) with developmental delay, speech impairment, clinodactyly, anisocoria, and unusual face shape. The other, a nonsense mutation in exon 4, occurred in a child and mother with typical features of KBG Syndrome.

## DISCUSSION

This study of British patients with a molecular genetic diagnosis of KBG syndrome is the largest to date, bringing the total number of published cases to >100. Our findings show that the characteristic facial phenotype of KBG syndrome can be recognized as indicated in previous reports [Herrmann et al., 1975; Smithson et al., 2000; Brancati et al., 2006; Skjei et al., 2007; Sirmaci et al., 2011; Youngs et al., 2011; Ockeloen et al., 2015]. On review of a panel of patient photographs, (Fig. 2) we identified in some but not all patients a triangular face, almond‐shaped palpebral fissures which may slant upwards, synophrys, prominent ears, a high nasal bridge and bulbous nasal tip, prominent cheekbones, a smooth philtrum with thin upper lip, prominent corners of the mouth, and facial hypertrichosis/low hairlines. Our impression from available data is that the features can evolve in childhood and become more obvious in time. We agree with other authors that while some KBG patients can be recognized by gestalt, others may resemble Cornelia de Lange syndrome (CDLS). Ansari et al. [2014] found that 3/163 CDLS‐like patients had *ANKRD11* mutations and Busa et al. [2015] reported on a French cohort of 20 mutation/deletion cases and commented that the facial features were reminiscent of CDLS. Parenti et al. [2016] also found an overlap in phenotyping and advised *ANKRD11* analysis for CDLS mutation negative patients. In our patients, we observed some facial similarities with CDLS, particularly the appearance of the eyebrows and eyelashes.

When we compared the facial phenotypes of patients in the two diagnostic pathways, we observed that in the targeted group the gestalt was present in a higher proportion of cases than in the DDD group, where it was only clear in three. In some patients, we considered that that the specific facial features were not present, consistent with the high ascertainment from the DDD study (19/32 cases).

In 22% of patients, we found a wide anterior fontanelle with delayed closure and speculate this may contribute to the triangular face shape in KBG syndrome. In some cases this was the presenting feature and led to consideration of cleidocranial dysostosis (MIM # 119600). Furthermore, delayed closure of the anterior fontanelle is one of the most distinctive features according to the Decipher gene browser view for *ANKRD11*. Other conditions with cranial involvement and a similarly broad forehead include Robinow syndrome (MIM # 180700) which was investigated in one patient in this study. Five of the patients were referred for investigation of possible craniosynostosis on the basis of the broad forehead and brachycephaly, but no evidence of fused cranial sutures was found. Although, patients with KBG syndrome can be relatively short in stature, we did not find evidence of a generalised skeletal dysplasia. The most consistent finding was brachydactyly with short tubular bones in the hands and feet radiographically. One of our patients (13) has been treated with growth hormone and some success has been reported elsewhere [Reynaert et al., 2015].

Our study clearly shows a predisposition for recurrent otitis media in KBG syndrome. This is important to identify and treat to reduce hearing loss and optimise speech development. We also identified palatal abnormalities/dysfunction in a quarter of patients, which may be missed without specialist assessment. Based on the findings of this study and others, we recommend some key measures for all patients with a new diagnosis of KBG syndrome (summarized in Table II) to assist pediatric and other colleagues involved in the care of patients.

**Table II:** Recommendations for Clinical Management of Patients With KBG Syndrome

Recommended investigations/follow up	
Diagnosis	Molecular testing *ANKRD11*
	Array CGH for 16q24 microdeletion if KBG phenotype and *ANKRD11* sequencing normal
Care coordinated by key clinician	Hospital or community paediatrician for children and GP for adults
Investigations to consider in all patients	Echocardiogram
	Palatal assessment with specialist team
	Hearing and vision assessment
	Specialist dental review
Referrals and on‐going surveillance that may be required	Neurology for seizures and movement disorders
	Cardiology if heart lesion identified
	Dietician for feeding issues
	Endocrinology for investigation of short stature if present
	Surgery for cryptorchidism and hernia
	Respiratory/sleep studies for apnoea
	ENT/audiology for recurrent otitis media and/or hearing loss
Management of learning and behaviour	Paediatric MDT assessment for developmental delay, ASD/ADHD and complex behaviour patterns
	Educational support where required
Investigations that may be indicated in individual patients	Skeletal survey/assessment of bone age
	Renal ultrasound
	MRI brain scan

All patients in this study had a mutation in *ANKRD11*, a member of a family of Ankyrin repeat containing cofactors described a decade ago [Zhang et al., 2004] which is expressed in a wide variety of tissues including the brain. It is implicated in activation of transcription by interaction with p160 coactivator and nuclear receptor complex by recruiting histone deacetylases [Sirmaci et al., 2011]. This may account for the pleiotropic effects of *ANKRD11* alterations. Subsequently, Ankrd11 has been shown to be a crucial chromatin regulator that controls histone acetylation and gene expression during neural development [Gallagher et al., 2015]. This provides a likely explanation for the widespread finding of variable learning difficulties and other cognitive dysfunction in our cohort. Yoda mice, heterozygous for a missense mutation in *ankrd11* show craniofacial anomalies, wide skulls, kyphoscoliosis, and reduced bone mineral density, but no other costovertebral anomalies or macrodontia [Sirmaci et al., 2011]. The precise role of *ANKRD11* in bone formation and modelling is also currently unknown.

Like other authors, we did not establish a predictive value for specific mutations, but it is possible that c.1903_1907del may be associated with a relatively mild phenotype with respect to ear infections and hearing loss and further work will be required to clarify this. This lack of genotype–phenotype correlation suggests that the variable nature of the KBG syndrome cannot be attributed to the position of truncation of the protein. This supports a common mechanism of pathogenicity in which truncating mutations in the C terminal domain would be expected to affect protein degradation [Walz et al., 2015]. Comparison of the phenotypes in patients 5–8 and 29–31 demonstrate intra‐familial variability and variable penetrance.

In addition to comparing the facial features, we revisited the whole phenotype in the two ascertainment groups. If sufficient characteristics are present, such as macrodontia in a child with developmental/speech delay, or learning difficulties, it is likely the diagnosis of KBG syndrome will be considered and TS initiated. It is clear from this study that the presentation is variable and the diagnosis can be missed [Ashraf et al., 2015], especially in infants and young children. For KBG, ES methodology revealed that a non‐specific *ANKRD11*‐related phenotype exists, as found for other conditions [Need et al., 2012]. To date (January 2016), in addition to those reported here, 14 further patients with a variant in *ANKRD11* have been identified by the DDD study (32 intragenic SNV/indels and one intragenic deletion in the first 4294 trios). This is consistent with our finding that the gestalt can be unclear and easily missed.

**Figure 3:**
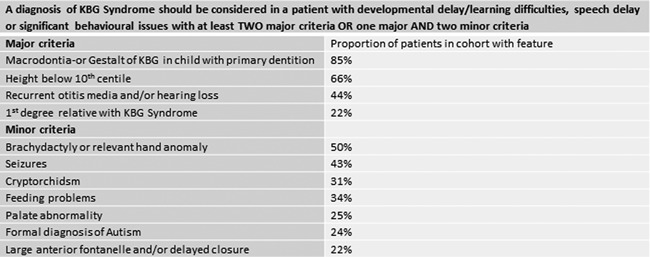
Diagnostic aid for KBG syndrome.

Skjei et al. [2007] suggested diagnostic criteria for KBG syndrome which required at least 4/8 features including delayed bone age and costovertebral anomalies to be present. Ockeloen et al. [2015] suggested that only 3/8 may be necessary based on their Dutch cohort. However, in our experience and in previous reports, many patients have not had an estimation of bone age or skeletal survey as they were not clinically indicated. Furthermore, despite consensus regarding the gestalt of KBG, our data demonstrate that the facial features can be very subtle or unrecognizable. We have therefore, devised a diagnostic aid for KBG syndrome (Fig. 3) which does not rely on the original features‐this would have identified all but one (patient 22 who is notably atypical in his overgrowth parameters) in our cohort. DDD has detected some mild cases with few features and colleagues who are experienced in clinical dysmorphology did not consider KBG syndrome in their differential diagnosis (personal communications).

## CONCLUSIONS

This study is the largest to date of patients with KBG syndrome. Detailed phenotyping has provided data on the range of physical features in this condition including wide anterior fontanelle with delayed closure, not previously described. We report on the significant range of developmental and behavioral problems that affect patients. The presentation of KBG may be non‐specific and the diagnosis made for the first time through ES; therefore, we recommend that *ANKRD11* is included in gene panels used to investigate developmental delay. KBG syndrome is emerging as a Mendelian cause of learning difficulties with important implications for genetic counselling given the autosomal dominant inheritance of this condition. We have devised a revised diagnostic aid for clinical use and suggest clinical investigations and follow up that are likely to improve patient care.

## Supporting information

Additional supporting information may be found in the online version of this article at the publisher's web‐site.

Supporting Data S1.

Supporting Data S2.

Supporting Data S3.

Supporting Data S4.

## Endnotes

1 It is acceptable to assess macrodontia subjectively for apparently increased maximum width of the tooth; or objectively by measuring mesiodistal tooth diameter (width) more than 2 SD above mean for age.[Carey et al., 2009]. In our cohort macrodontia was assessed subjectively by the referring clinician and then again on review of the images.
