# Supplementary figures and images for: Clinical and genetic aspects of KBG syndrome
Source: Am J Med Genet A. 2016 Sep 26;170(11):2835–46. doi: 10.1002/ajmg.a.37842 (PMC5435101; doi:10.1002/ajmg.a.37842)

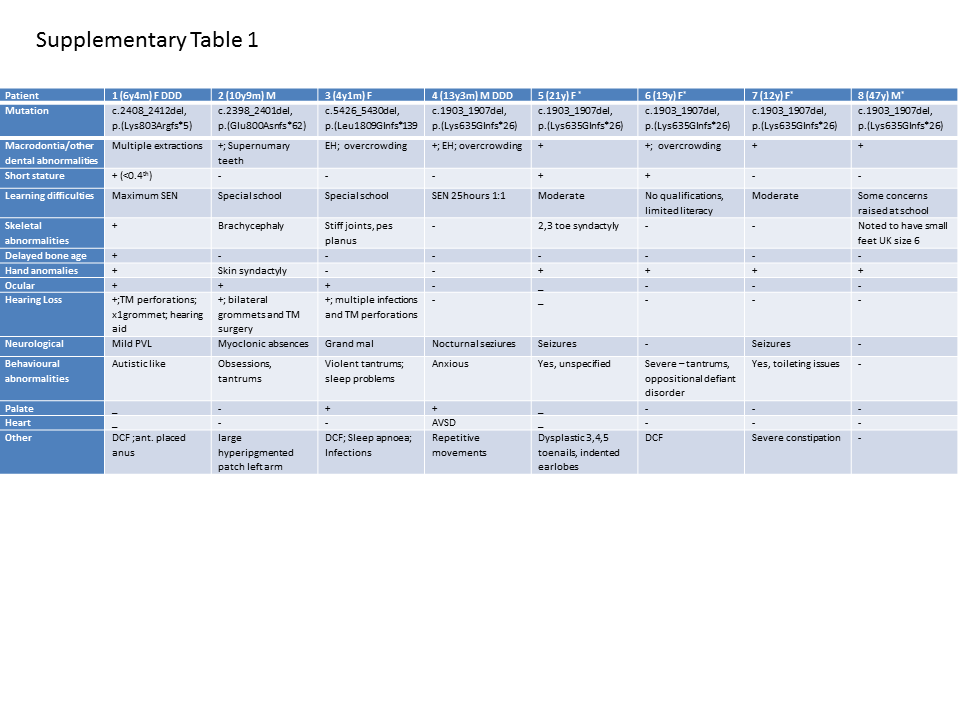

Supplement: Supplementary file 1 — Supporting Data S1. [file AJMG-170-2835-s001.tif]

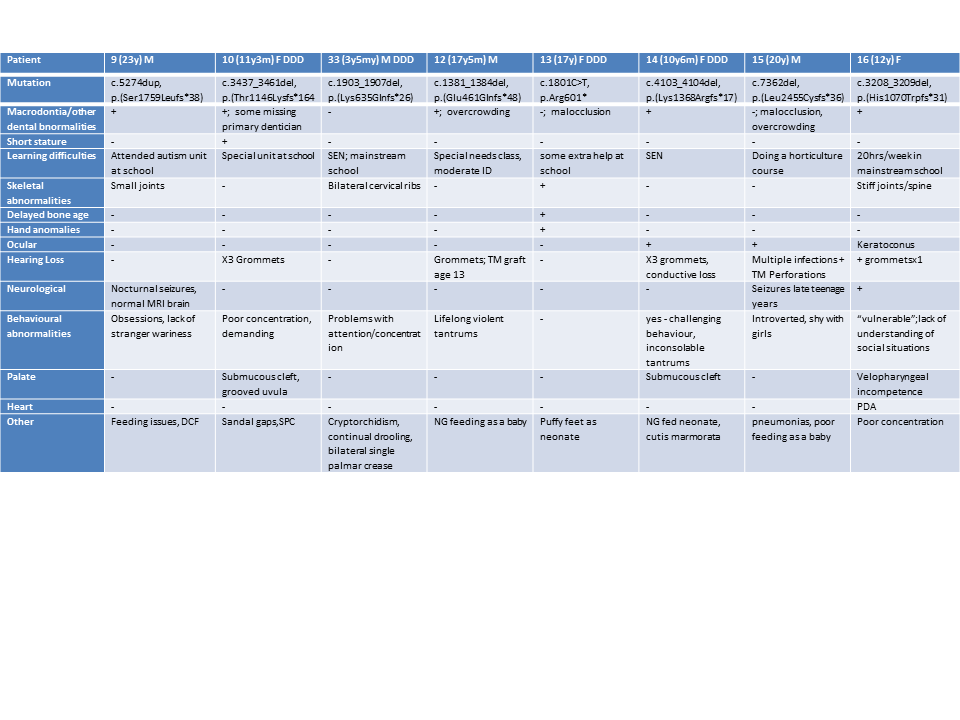

Supplement: Supplementary file 2 — Supporting Data S2. [file AJMG-170-2835-s002.TIF]

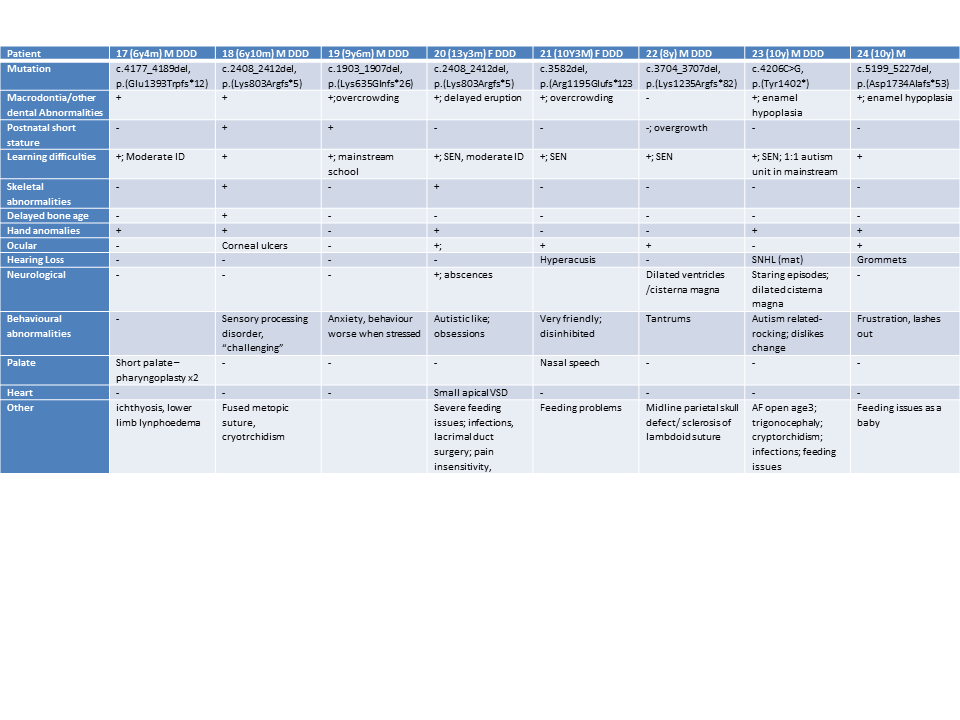

Supplement: Supplementary file 3 — Supporting Data S3. [file AJMG-170-2835-s003.TIF]

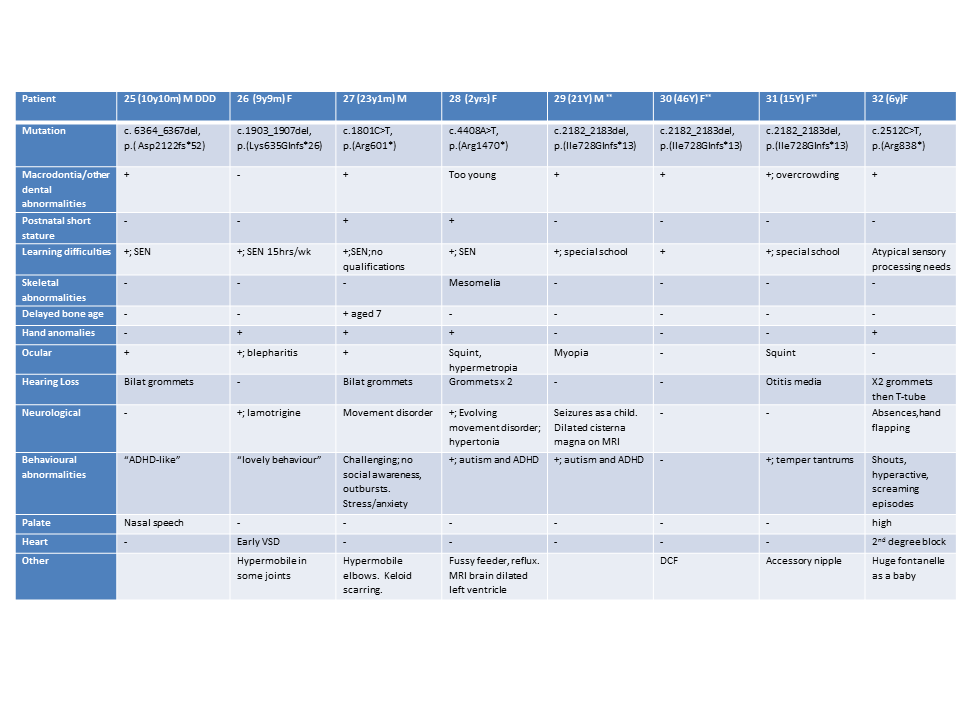

Supplement: Supplementary file 4 — Supporting Data S4. [file AJMG-170-2835-s004.TIF]
